# Sensing Mechanisms: Calcium Signaling Mediated Abiotic Stress in Plants

**DOI:** 10.3389/fpls.2022.925863

**Published:** 2022-06-13

**Authors:** Tongfei Xu, Junfeng Niu, Zhonghao Jiang

**Affiliations:** ^1^College of Life Sciences and Oceanography, Shenzhen University, Shenzhen, China; ^2^Guangdong Provincial Key Laboratory for Plant Epigenetics, College of Life Sciences and Oceanography, Shenzhen University, Shenzhen, China

**Keywords:** abiotic stress, sensing mechanisms, Ca^2+^ signaling, sensor, transduction

## Abstract

Plants are exposed to various environmental stresses. The sensing of environmental cues and the transduction of stress signals into intracellular signaling are initial events in the cellular signaling network. As a second messenger, Ca^2+^ links environmental stimuli to different biological processes, such as growth, physiology, and sensing of and response to stress. An increase in intracellular calcium concentrations ([Ca^2+^]_i_) is a common event in most stress-induced signal transduction pathways. In recent years, significant progress has been made in research related to the early events of stress signaling in plants, particularly in the identification of primary stress sensors. This review highlights current advances that are beginning to elucidate the mechanisms by which abiotic environmental cues are sensed *via* Ca^2+^ signals. Additionally, this review discusses important questions about the integration of the sensing of multiple stress conditions and subsequent signaling responses that need to be addressed in the future.

## Introduction

Plants encounter various abiotic stresses in their natural environment, for example, mechanical stimuli, drought, flood, cold, and salinity stress. These adverse conditions often limit plant growth and crop productivity worldwide (Zhang et al., 2018b). To survive, plants, which are sessile organisms, must detect the nature and strength of environmental stimuli, transduce these signals into intracellular signaling and activate appropriate physiological responses (Kissoudis et al., 2014; Zhang et al., 2022). The perception of environmental stress and the subsequent transduction of stress signals are initial events in the adaptation of all organisms to stresses in their environment (Lamers et al., 2020). Therefore, how plants sense abiotic stress signals, transduce them into cellular signaling and subsequently adapt to adverse environment is a fundamental and significant biological question.

Typically, a primary abiotic stress sensor is required to perceive external environmental changes; in response to such changes, the sensor remodels signal transduction pathways and initiates appropriate responses that allow the plant to adapt to the stress condition. Thus, perception of the external environment by a sensor is the earliest step in the conversion of external stimuli into cellular signals by plants.

Calcium (Ca^2+^), an indispensable second messenger, is considered to be a critical element by which plants modulate a complicated network of signaling pathways to respond to various abiotic stresses (Hetherington and Brownlee, 2004; Pandey et al., 2004; Dodd et al., 2010; Yuan et al., 2014). The increase in intracellular calcium concentrations ([Ca^2+^]_i_) is one of the earliest signaling events when plants are challenged with abiotic stimuli, and the resulting Ca^2+^ signaling regulates many processes in plants, including transcriptional regulation and subsequent physiological as well as developmental responses (Dodd et al., 2010; Kudla et al., 2010; Cao et al., 2017; Aldon et al., 2018). Bioluminescence-based aequorin technology for the detection of [Ca^2+^]_i_ oscillations has been reported, and this technology uses the aequorin system to detect abiotic stimuli-induced Ca^2+^ signals (Knight et al., 1991, 1997). The results obtained with this approach have suggested that Ca^2+^ is an important second messenger for understanding plant-abiotic stimulus interactions (Van Zelm et al., 2020).

In the resting state, [Ca^2+^]_i_ is maintained within a range of 50–200 nM (Berridge et al., 2000; Mehlmer et al., 2012; Lee and Seo, 2021). However, this concentration increases by approximately 10-fold and reaches millimolar levels upon stimulation (Lee and Seo, 2021). Transient increases in Ca^2+^ levels are caused by Ca^2+^ influx from the extracellular space or Ca^2+^ release from organelles through Ca^2+^ channels and transporters in membranes (Rentel and Knight, 2004; Kudla et al., 2010; Batistic and Kudla, 2012; Stael et al., 2012). Various stresses activate different Ca^2+^ channels and trigger [Ca^2+^]_i_ increase in cells, and these increases exhibit tissue-specific and stress-specific differences in peak amplitude, peak oscillation pattern, and wave propagation. This stress-specific phenomenon is known as a “Ca^2+^ signature,” which leads to the activation of downstream events, such as stress-responsive gene expression and protein interactions (Jeworutzki et al., 2010; Stael et al., 2012; Steinhorst and Kudla, 2013; Seybold et al., 2014). Despite several potential stress sensors functioning as Ca^2+^-permeable cation channels (e.g., several ion channels that directly sense membrane tension), the link between abiotic stress sensors and the Ca^2+^ influx channels that are regulated by these sensors is still largely unknown. Here, we mainly discuss recent findings related to Ca^2+^ signaling to reveal the mechanisms by which plants sense stressful environment; these findings have advanced our understanding of plant sensory mechanisms.

## Mechanical Stimuli

Responses to environmental stress are essential features of plant behavior (Braam, 2005). A fundamental characteristic of plants is their ability to sense and respond to mechanical stimuli, such as touch, gravity, and flexure (Trewavas and Knight, 1994; Braam, 2005; Nakagawa et al., 2007). When *Arabidopsis* plants that express a Ca^2+^-sensitive aequorin are exposed to touch and gravistimulation, the recombinant plants emit light immediately (Plieth and Trewavas, 2002; Toyota et al., 2008), suggesting that mechanical stimuli influence membrane-localized Ca^2+^-permeable channels and immediately trigger a transient increase in [Ca^2+^]_i_; this is an early event in the response to mechanical stimuli, and it subsequently influences plant growth and development. In addition, osmotic stress (including both hyperosmotic stress caused by drought and hypoosmotic shock) also influences membrane tension and triggers a [Ca^2+^]_i_ increase by activating certain Ca^2+^-permeable channels, allowing plant cells to perceive changes in the physical properties of the membrane (Gong et al., 2020).

To date, five families of mechanosensitive channels have been reported in plants: mechanosensitive channel of small conductance (MscS)-like proteins (MSLs), Mid1-complement activity protein channels (MCAs), two-pore potassium channels (TPKs), Piezo channels (PZO), and reduced hyperosmolality induced [Ca^2+^]_i_ increase channels (OSCA; Haswell and Meyerowitz, 2006; Nakagawa et al., 2007; Haswell et al., 2008; Coste et al., 2010; Yamanaka et al., 2010; Maathuis, 2011; Yuan et al., 2014).

**Figure 1:**
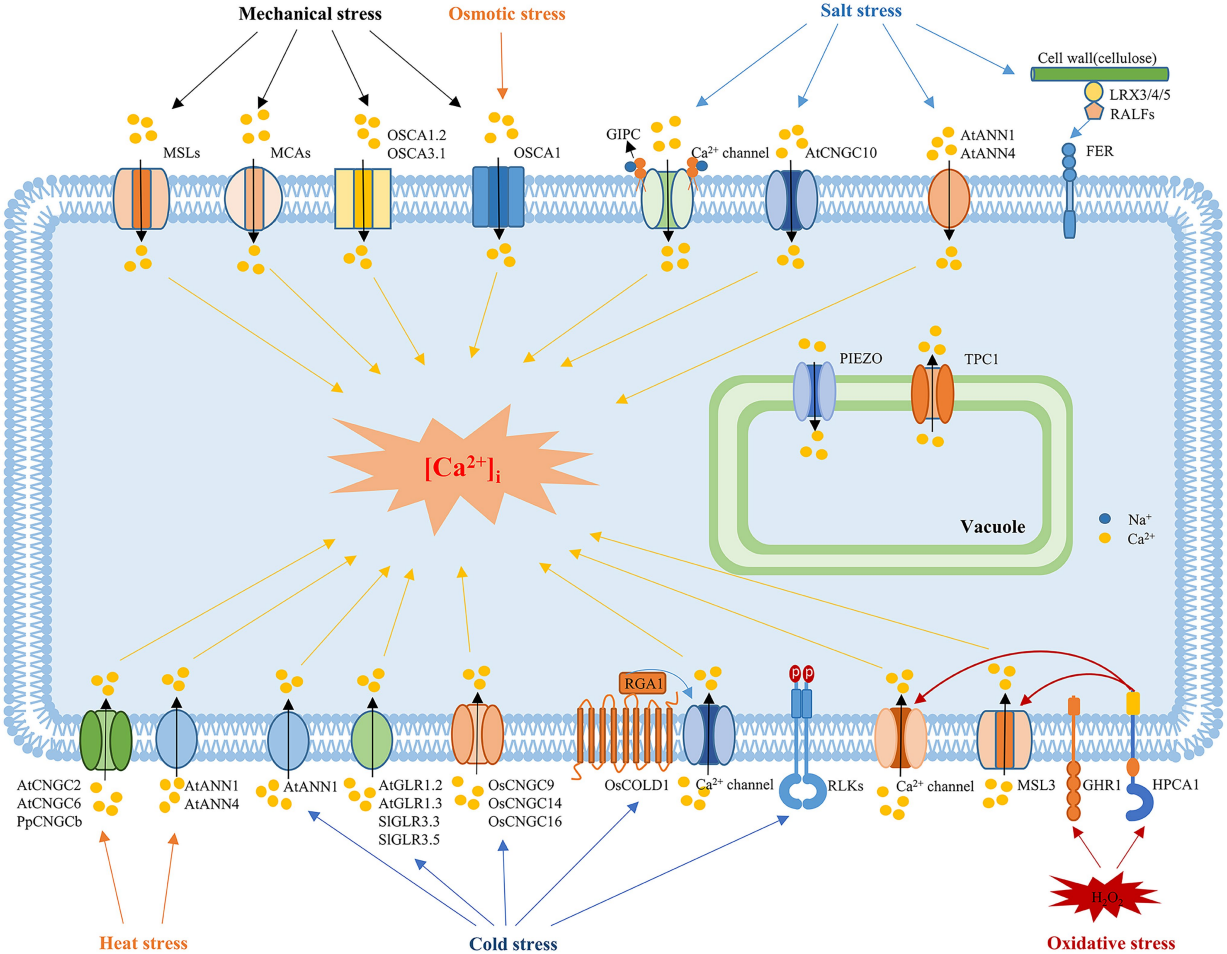
Mechanisms underlying the sensing of abiotic stress in plants. In response to mechanical stimuli, mechanosensitive channels, such as MSLs, MCA, Piezos, and OSCAs, are activated to induce Ca^2+^ influx. MCAs and OSCA are plasma membrane mechanosensitive cation channels, while Piezo is located in the vacuole membrane. OSCA1 is a plasma membrane protein that functions as a hyperosmolality-gated calcium-permeable channel that induces the initial increase in [Ca^2+^]_i_ during osmotic stress. Salt stress is sensed by the binding of monovalent cations to the negatively charged GlcA of the GIPC sphingolipids. Upon Na^+^ binding, the GIPC-gated unknown Ca^2+^ channel is activated to trigger the Ca^2+^ influx. ANN1, ANN4, and CNGC10 are also involved in the salt-induced Ca^2+^ influx. In addition, the cell wall-localized leucine-rich repeat extensins LRX3, LRX4, and LRX5, RALFs and the receptor-like kinase FER also participate in sensing salt stress signals. The vacuolar ion channel TPC1 is involved in systemic salt and Ca^2+^ signaling. Environmental stresses trigger tissue-specific and stress-specific [Ca^2+^]_i_ increases, which are known as “Ca^2+^ signatures.” The crucial protein in sensing cold stress in rice is COLD1. COLD1 interacts with RGA1 in response to cold temperatures, resulting in increased GTPase activity, which activates an unknown calcium influx channel. Plasma membrane-localized RLCK plays a negative role in regulating freezing tolerance. In addition, plant CNGCs, such as CNGC9, CNGC14, and CNGC16 in rice, are involved in cold sensing, while CNGC2 and CNGC6 in *Arabidopsis* and CNGCb in *Physcomitrella* mediate the heat-induced [Ca^2+^]_i_ increase. Glutamate receptors, including GLR1.2 and GLR1.3 in *Arabidopsis* and GLR3.3 and GLR3.5 in tomato plants, also play roles in cold sensing and tolerance. ANN1 and ANN4 are involved in the heat-induced [Ca^2+^]_i_ increase and tolerance to heat, while ANN1 is also involved in the cold-induced Ca^2+^ influx. H_2_O_2_ perception causes oxidation of HPCA1, and oxidative modification of HPCA1 might act alone or with a coreceptor to phosphorylate and activate an unknown Ca^2+^ channel to trigger Ca^2+^ influx. GHR1, a plasma membrane receptor-like kinase, may also sense H_2_O_2_ signals and regulate ABA signaling and stomatal closure in guard cells. MSL3 and TPC1 are involved in systemic ROS and Ca^2+^ signaling.

MCA proteins were identified as PM-localized Ca^2+^-permeable mechanosensitive ion channels that regulate mechanoresponsive Ca^2+^ influx (Nakagawa et al., 2007; Figure 1). AtMCA1 and AtMCA2 are two paralogous MCA genes in *Arabidopsis* (Yamanaka et al., 2010; Nishii et al., 2021). Mca1-knockout seedlings have reduced amplitudes and very slow [Ca^2+^]_i_ increases (Nakano et al., 2021). Ectopic overexpression of MCA proteins increases Ca^2+^ uptake in *Arabidopsis* seedlings as well as in cultured rice cells and enhances the hypo-osmotic shock-induced increase in [Ca^2+^]_i_ (Nakagawa et al., 2007; Kurusu et al., 2012a,b). In addition, MCA1 was first reported to induce a gravistimulation-induced [Ca^2+^]_i_ increase under the condition of 1–5 g gravitational acceleration (Nakano et al., 2021). Recently, the mechanisms underlying MCA activation have been elucidated. Yoshimura et al. purified and reconstituted MCA1 and MCA2 into artificial liposomal membranes and examined their Ca^2+^ permeability properties. They found that a C-terminally truncated form of the MCA2 protein that acts as a Ca^2+^-permeable and mechanosensitive channel directly senses membrane tension to regulate channel opening (Yoshimura et al., 2021).

Using calcium imaging, *Arabidopsis thaliana* PIEZO1 has been shown to exhibit the conserved function of the mammalian PIEZO mechanosensitive ion channel, and this channel responds to a mechanical stimulus with transiently increasing Ca^2+^ levels (Mousavi et al., 2021). Evidence has shown that plant PIEZO1 is expressed in the columella and lateral root cap cells of the root tip, which experience robust mechanical strain during root growth. Deleting PIEZO1 from the whole plant significantly reduced the ability of roots to penetrate denser barriers compared to that of the wild-type plants. The root tips of *piezo1* mutant plants exhibited decreased transient changes in Ca^2+^ levels in response to mechanical stimulation, supporting a role of plant PIEZO1 in root mechanotransduction. Recently, a very interesting finding was reported that plant PIEZO was found to be localized to the vacuole membrane, where it plays an important role in regulating the formation of vacuole morphology and inducing [Ca^2+^]_i_ oscillation (Radin et al., 2021; Figure 1).

Based on forward genetic screening to isolate EMS-mutagenized aequorin-expressing *Arabidopsis* seedlings, OSCA1 was the first potential osmosensor to be identified (Yuan et al., 2014). Mutation in OSCA1 results in a decrease in Ca^2+^ accumulation in guard cells and root cells, as well as a lack of transpiration regulation and reduced root growth in response to osmotic stress. OSCA1 was identified as a hyperosmolality-gated Ca^2+^-permeable channel, and it is responsible for the initial increase in free Ca^2+^ concentrations upon the sensing of osmotic stress (Yuan et al., 2014; Figure 1). The activation of these channels has been hypothesized to be caused by mechanical forces on the cell membrane or cell wall generated by osmotic stress (Gong et al., 2020). Phylogenetic analysis demonstrated that *Arabidopsis* has 15 homologs of OSCA1, suggesting that the sensing of hyperosmotic conditions could be mediated by a redundant family of Ca^2+^ channels (Liu et al., 2018). Using cryo-electron microscopy (cryo-EM), three groups have separately characterized the structures of OSCA1.1, OSCA1.2, and OSCA3.1, and these groups found that the OSCA proteins belong to a new class of mechanosensitive ion channels with structural similarities to mammalian TMEM16-family proteins (Jojoa-Cruz et al., 2018; Liu et al., 2018; Zhang et al., 2018a; Figure 1).

## Salt Stress

Soil salinization is a severely adverse abiotic factor that affects seed germination, crop growth, and productivity (Zhao et al., 2020). In contrast to animals, sodium is not an essential element to most plants, and at high concentrations, it is very detrimental to plant growth. High concentrations of Na^+^ and Cl^−^ in soil induce both osmotic stress and ionic stress. When exposed to high levels of Na^+^, large, low-affinity Na^+^ fluxes occur that are mediated by HKT-type carriers (Garciadeblas et al., 2003; James et al., 2011) and nonselective ion channels (NSCCs), including glutamate receptor-like (GLR) and cyclic nucleotide-gated channels (CNGCs; Amtmann and Sanders, 1999).

By performing a forward genetic screen, an *Arabidopsis* monocation-induced [Ca^2+^]_i_ increases 1 (moca1) mutant was isolated successfully, and this mutant exhibits decreased Na^+^-induced increases in [Ca^2+^]_i_ and is hypersensitive to growth inhibition by Na^+^ (Jiang et al., 2019). The lack of MOCA1 activity also impairs the membrane surface potential and, notably, the activity of the Na^+^/H^+^ antiporter SOS1, making the mutant plants more sensitive to salt than the wild-type plants. Thus, MOCA1 represents one of the missing links between salt perception and SOS pathway activation. MOCA1 was identified as a glucuronosyltransferase that transfers a negatively charged GlcA to inositol phosphorylceramide (IPC) to form glycosylinositol phosphorylceramide (GIPC) sphingolipids in the external layer of the plasma membrane, and it is essential for NaCl-triggered depolarization of the cell-surface potential (Jiang et al., 2019). The study found that *moca1* mutant plants contained lower levels of GIPCs but higher levels of IPCs than wild-type plants (Jiang et al., 2019). Since IPCs do not contain a negatively charged head, there are fewer monovalent cation binding sites on the membranes of *moca1* mutant plants than on the membranes of wild-type plants, which is consistent with the previously described strong Na^+^-binding properties of GIPCs (Markham et al., 2006). Regarding the Na^+^ perception mechanism, Na^+^ directly binds to GIPCs and presumably regulates Ca^2+^ influx channels, providing the molecular basis for Na^+^ sensing in plants (Figure 1). However, the exact mechanism of GIPC-mediated Ca^2+^ influx remains unknown as long as the Ca^2+^ channel involved remains unidentified (Steinhorst and Kudla, 2019). In addition, downstream of GIPCs, the Ca^2+^ wave speed is altered in the *tpc1* (two-pore channel 1) mutant, indicating that TPC1 mediates Ca^2+^ release from the vacuole and facilitates the propagation of Ca^2+^ waves (Choi et al., 2014). A putative glycosyltransferase QUA1, required for normal pectin synthesis and cell adhesion, was reported to regulate [Ca^2+^]_i_ in response to salt stress in *Arabidopsis* (Zheng et al., 2017).

Luminescence-based detection of [Ca^2+^]_i_ showed that the Ca^2+^-permeable transporter AtANNEXIN1 (AtANN1) is required for NaCl-activated Ca^2+^ influx through the plasma membrane in root epidermal cells. The loss-of-function mutation of AtANN4 also disrupts the salt-induced [Ca^2+^]_i_ increases in *Arabidopsis* (Laohavisit et al., 2013; Ma et al., 2019). In addition to AtANNs, CNGC10 was reported to negatively regulate salt tolerance in *Arabidopsis* (Jin et al., 2015). It would be interesting to investigate whether there is a direct functional association between GIPC and AtANN1, AtANN4, or CGNC10. In addition, the cell wall is involved in salt sensing. A receptor-like kinase FERONIA (FER) possibly perceives salt-induced changes in the cell wall. Mutation of FER results in decreased salt-induced Ca^2+^ signaling and increased sensitivity to high salinity (Feng et al., 2018; Figure 1). Recent studies have shown that the cell wall-localized leucine-rich repeat extensins LRX3, LRX4, and LRX5 and the secretory peptide RALFs participate in sensing salt stress signals along with FER (Figure 1). However, the mechanism of the initial sensing of salt-induced changes in the cell wall by LRXs remains unknown (Zhao et al., 2018).

## Temperature Stress

Temperature is one of the major environmental factors that affect plant growth and development. Extreme temperatures induce a series of biochemical, physiological, and morphological changes in plants and often reduce plant productivity (Cui et al., 2020a). Cold stress decreases the fluidity of cellular membranes and causes membrane rigidification in plant cells, which also disrupts the stability of proteins or protein complexes and decreases the activities of enzymes (Orvar et al., 2000; Zhu, 2016; Gong et al., 2020). To survive under extreme temperatures, plants must be able to perceive temperature signals and transduce them to the downstream signaling pathways, subsequently activating appropriate defense mechanisms (Guo et al., 2018).

Cold sensing is followed by the generation of secondary messengers, including Ca^2+^ (Yan et al., 2006). After exposure of both plants and animals to cold, [Ca^2+^]_i_ is increased very rapidly *via* Ca^2+^ channels, which is thought to be one of the earliest events in cold stress signaling (Knight et al., 1991; Ding et al., 2019). Cold stress induces transient Ca^2+^ influx into the cell cytoplasm, and repeated cold treatment can induce repetitive transient Ca^2+^ influxes (Krebs et al., 2012). In mammals, the transient receptor potential (TRP) superfamily of cation channels is involved in thermosensation (Waszczak et al., 2018). However, no TRP orthologs have been identified in plants. How Ca^2+^ signals are involved in cold perception in plants is unclear. Ma et al. identified a transmembrane protein named COLD1 (chilling-tolerance-divergence 1), which was the first potential cold sensor to be identified in rice. Overexpression of COLD1 significantly improves survival rates after chilling treatment, whereas mutants with deficiency or decreased expression of COLD1 are sensitive to chilling stress (Ma et al., 2015). These results suggest that COLD1 is an important component of the regulation of chilling tolerance in rice. Further study showed that COLD1 regulates G protein signaling by interacting with rice G protein subunit 1 (RGA1) to increase GTPase activity under conditions of cold stress. Furthermore, COLD1-RGA1 form a complex to trigger a cold-induced increase in [Ca^2+^]_i,_ leading to the activation of the cold tolerance response (Figure 1). However, the molecular mechanism by which COLD1 senses cold stress and COLD1 activates Ca^2+^ channels remains unclear. It would be interesting to determine whether COLD1 itself functions as a temperature-regulated membrane ion channel and how COLD1 acts as a cold sensor to trigger cold-induced Ca^2+^ influx in plants.

In addition, cold can decrease the fluidity of cellular membranes (Zhu, 2016). This change in membrane fluidity may be sensed by membrane-localized proteins, such as Ca^2+^ channels and receptor-like kinases (RLKs; Zhu, 2016, Gong et al., 2020). Several RLKs have been reported to play critical roles in regulating cold responses. CRLK1 and CRLK2 are two calcium/calmodulin-regulated receptor-like cytoplasmic kinases (RLCKs) that positively regulate cold tolerance (Yang et al., 2010). Recently, a plasma membrane-localized RLCK, cold-responsive protein kinase 1 (CRPK1), was found to play a negative role in regulating cold tolerance (Liu and Howell, 2016; Figure 1). Plant CNGCs are involved in thermal sensing and thermotolerance (Finka et al., 2012). In rice, loss-of-function mutant of OsCNGC9 is defective in cold-induced Ca^2+^ influx, which make plants more sensitive to cold treatment; these results suggest that OsCNGC9 could positively regulate chilling tolerance (Wang et al., 2021). Additionally, the loss of either OsCNGC14 or OsCNGC16 reduced or abolished the [Ca^2+^]_i_ escalation by either heat or chilling stress, indicating that OsCNGC14 and OsCNGC16 modulate Ca^2+^ signaling in response to temperature stress (Cui et al., 2020b; Figure 1). A previous study reported that CNGCb in *Physcomitrella* and its *Arabidopsis* ortholog AtCNGC2 mediate heat-induced [Ca^2+^]_i_ increases, enhancing plant survival at high temperatures (Finka et al., 2012; Figure 1). In addition, AtCNGC6 in *Arabidopsis* is also involved in heat-induced induced [Ca^2+^]_i_ increases (Gao et al., 2012; Figure 1). In addition to CNGCs, AtANNs are involved in temperature dependent Ca^2+^ influx. A recent study reported that loss-of-function mutations in AtANN1 significantly impair cold-induced Ca^2+^ influx and reduce tolerance to cold stress in *Arabidopsis* (Liu et al., 2021; Figure 1). Moreover, AtANN1 and AtANN4 are also involved in heat-induced [Ca^2+^]_i_ increases and consequently heat tolerance in *Arabidopsis* (Liao et al., 2017; Figure 1). Additionally, GLR could act as sensors and mediators for temperature in plants. Plants with loss-of-function mutations in AtGLR1.2 and AtGLR1.3 are sensitive to cold stress, while AtGLR1.2 and AtGLR1.3 increase cold tolerance by regulating jasmonate signaling in *Arabidopsis* (Zheng et al., 2018; Figure 1). GLR3.3 and GLR3.5 are involved in cold tolerance in tomato plants (Li et al., 2019; Figure 1).

## Oxidative Stress

Reactive oxygen species (ROS) play a key role in plant cell signaling. Plant cells generate various ROS, including hydrogen peroxide (H_2_O_2_), which is produced extracellularly in response to external stresses and internal cues. Extracellular hydrogen peroxide (eH_2_O_2_) plays a vital role in many physiological processes during the lifecycle of plants, such as root development, pollen tube growth, organ wilting, and responses to biotic and abiotic stress (Waszczak et al., 2018).

It has been reported that the H_2_O_2_ signal can be sensed by Guard Cell Hydrogen Peroxide-Resistant1 (GHR1), which regulates ABA signaling and stomatal closure in guard cells (Hua et al., 2012; Figure 1). Additionally, GHR1 is also involved in stomatal responses to high CO_2_ concentrations, activating guard cell anion channel slow anion channel1 (SLAC1) and stimulating stomatal closure (Horak et al., 2016; Sierla et al., 2018). In addition, the influx of eH_2_O_2_ into the cytosol *via* aquaporin channel proteins has also been reported, but whether and how plants perceive eH_2_O_2_ at the cell surface is unknown. Although little is known about the initial sensing of eH_2_O_2_, the fact that eH_2_O_2_ triggers an influx of Ca^2+^ is much better defined and is thought to be involved in H_2_O_2_ sensing and signaling. Interestingly, Ca^2+^ was also found to bind respiratory burst oxidase homolog protein D (RBOHD) EF-hands and promotes H_2_O_2_ which then subsequently activates Ca^2+^ channels in response to a range of abiotic stresses (Ogasawara et al., 2008; Miller et al., 2009; Suzuki et al., 2011).

By using a forward genetic screen based on Ca^2+^ imaging, Wu et al. isolated hydrogen peroxide-induced Ca^2+^ increase (*hpca*) mutants in *Arabidopsis* and showed that HPCA1 encodes a leucine-rich repeat receptor kinase that localizes to the cell surface. It was the first molecular component found to be responsible for sensing eH_2_O_2_ (Pei et al., 2000; Wu et al., 2020). The *hpca1* mutant did not display growth or developmental phenotypes, but eH_2_O_2_- and ABA-insensitive phenotypes were observed in terms of stomatal closure. In addition, the *hpca1* mutant exhibits decreased eH_2_O_2_-induced Ca^2+^ influx, indicating that HPCA1-mediated activation of Ca^2+^ channels is critical for eH_2_O_2_ signaling. Further study demonstrated that HPCA1 can be activated by eH_2_O_2_ *via* the covalent modification of its extracellular cysteine residues, which leads to autophosphorylation of HPCA1 and its function as an eH_2_O_2_ sensor to induce Ca^2+^ influx (Figure 1).

A recent research finding suggested that MSL3 could function downstream of HPCA1, indicating that HPCA1 is required for systemic ROS and Ca^2+^ cell-to-cell signaling and plant acclimation to stress (Fichman et al., 2022). In addition, ROS also stimulate the vacuolar ion channel TPC1 to release Ca^2+^ from the vacuole, allowing substantial propagation of systemic Ca^2+^ waves (Evans et al., 2016).

## Conclusion and Perspectives

As sessile organisms, plants cannot escape from environmental stress. It is very important for plants to accurately and specifically recognize environmental signals and initiate the correct responses. In the last decade, some major advances have been made in the elucidation of mechanisms underlying abiotic stimulus perception, such as the role of OSCA1 in response to osmotic stress, the role of COLD1 in sensing cold stress, the role of GIPCs as monovalent cation sensors, and the role of HPCA1 as an eH_2_O_2_ sensor. At present, many gaps remain in our understanding of plant stress sensing. Identifying stress sensors is an important goal for studying abiotic stress responses in plants, but overcoming genetic redundancy and lethality remains a challenge.

The increase in [Ca^2+^]_i_ is one of earliest signaling events when plants are challenged with abiotic stimuli. Ca^2+^ influx occurs within a few seconds after the perception of environmental stress. Different sensors sense specific stressors to directly or indirectly activate Ca^2+^ channels and mediate the influx of Ca^2+^. However, the identity of the Ca^2+^ channel involved in signal sensing and transduction and how environmental signals activate Ca^2+^ channels remain largely unknown. Therefore, the identification of sensing mechanisms is an indispensable step in the elucidation of cellular signaling pathways that participate in the response to environmental stress and how they lead to appropriate responses. Furthermore, more attention should be given to the functional coordination of Ca^2+^ channels in different cellular organelles because [Ca^2+^]_i_ could be complicated by the interplay of PM-localized influx channels and organelle-specific efflux channels.

Molecular genetics methods and various bioimaging techniques have contributed greatly to our current knowledge. With recent advances in bioimaging techniques and genetic methods, utilizing Ca^2+^ imaging-based forward genetic screens may be an effective way to reveal the mechanisms underlying stress sensing and potential Ca^2+^ channels in the future. These advances in understanding mechanisms underlying abiotic stimulus perception demonstrate that genetic screens based on “imaging technologies” represent the new standard for discovering new genes involved in important signaling pathways. In addition to bioluminescence approaches, fluorescence-based Ca^2+^ indicators have potential for single-cell analyzes (Krebs et al., 2012), but they are also mainly used at the tissue level. Fluorescence-based Ca^2+^ indicators can be broadly categorized into single fluorescent proteins (such as green fluorescent GCaMPs/G-GECOs and red fluorescent R-GECOs; Diao et al., 2018; Vigani and Costa, 2019; Luo et al., 2020) and dual-fluorescent protein indicators (such as FRET-based yellow cameleons; Allen et al., 1999; Walia et al., 2018; Vigani and Costa, 2019). Note that ratiometric Ca^2+^ indicators can compensate for variations in fluorescence readouts due to differences in expression levels between different cell and tissue types. It is clear that the increasing development of plant Ca^2+^ indicators should provide a fruitful foundation for novel discoveries.

Although several stress sensors have been reported recently, their physiological functions and biochemical sensing mechanisms remain unclear. Furthermore, because plants are exposed to multiple abiotic stresses simultaneously in the natural environment, determining stress-specific sensing mechanisms at the molecular level is important. Thus, to improve our understanding of abiotic stress resistance in plants, more attention should be given to the perception of multiple abiotic stresses by plants in the future.

In addition to sensing mechanisms, a great deal of responses and crosstalk are observed in downstream signaling pathways, which could lead to additive, synergistic or antagonistic effects, resulting in either enhanced or compromised stress resistance. However, since most current studies have focused on a single stress condition, the mechanisms by which plants respond to combined stresses remains largely unpredictable. Thus, revealing the mechanisms underlying plant perception and responses to multiple stresses at the molecular level will be necessary to gain knowledge about the integration of stress-induced signaling pathways, which is a promising direction for future research. Knowledge of how plants perceive and respond to stress will allow us to answer fundamental questions of how plants convert abiotic stress into electrochemical signals and will allow us to understand important biological processes by which how plants grow and develop to tolerate stressful environment.

## Author Contributions

TX, JN, and ZJ conceived the idea and wrote the manuscript. All authors contributed to the article and approved the submitted version.

## Funding

This work was supported by grants from Natural Science Foundation of Guangdong Province (2022B1515020040) and Shenzhen Peacock Plan (KQTD2017032715165926).

## Conflict of Interest

The authors declare that the research was conducted in the absence of any commercial or financial relationships that could be construed as a potential conflict of interest.

## Publisher’s Note

All claims expressed in this article are solely those of the authors and do not necessarily represent those of their affiliated organizations, or those of the publisher, the editors and the reviewers. Any product that may be evaluated in this article, or claim that may be made by its manufacturer, is not guaranteed or endorsed by the publisher.
